# Optimization of enzyme-linked immunosorbent assay kit protocol to detect trimethylamine N-oxide levels in humans

**DOI:** 10.17179/excli2022-5617

**Published:** 2023-02-20

**Authors:** Zinnet Sevval Aksoyalp, Betül Rabia Erdogan, Saliha Aksun

**Affiliations:** 1 Izmir Katip Celebi University, Faculty of Pharmacy, Department of Pharmacology, Izmir, TR; 2 Izmir Katip Celebi University, Faculty of Medicine, Department of Medical Biochemistry, Izmir, TR

**Keywords:** trimethylamine N-oxide, enzyme-linked immunosorbent assay, methods, investigative techniques, standardization

## Abstract

The serum level of trimethylamine N-oxide (TMAO), a gut microbiota metabolite associated with diabetes, cancer, inflammatory and neurological diseases, can be determined by the micro-enzyme-linked immunosorbent assay (ELISA) method. However, we had problems obtaining accurate standard curves with the original kit protocol from Bioassay Technology Laboratory. We aimed to acquire proper standard curves by modifying the kit protocol in this study. First, we evaluated the human TMAO ELISA kit protocols and other human ELISA kits. We maintained the incubation times longer and increased the wash cycle. Moreover, we incubated the standards containing biotinylated antibody in the wells alone. Then we washed the wells and added streptavidin-HRP for the second incubation step. The data of original and modified ELISA kit protocol were analyzed with Student's t-test. We measured higher absorbance with lower standard solution concentration in experiments that followed the original kit protocol. After investigating other human TMAO ELISA kits, we noticed that the SunRed Biotechnology Company and MyBioSource companies suggested similar protocols to the Bioassay Technology Laboratory company. The ELK Biotechnology ELISA protocol was different from others. However, since there is no biotinylated antibody in the standard solution in the ELK biotechnology kit, we changed some steps by examining other human ELISA protocols from different companies. After performing the modified protocol, we found that the absorbances of the standard solutions were consistent with their concentrations, and we obtained an accurate standard curve. Higher R^2 ^values and lower absolute difference of standard concentrations were found in the modified kit protocol. The human TMAO ELISA protocol, which we modified in this study, will enable researchers to obtain more reliable results and prevent them from failing time and resources.

## Introduction

Trimethylamine N-oxide (TMAO) is a water-soluble amine oxide small compound with a molecular formula (CH_3_)_3_NO (75.1 Da) (Liu and Dai, 2020[21]). Food sources such as choline, lecithin and carnitine are converted to TMA by the TMA lyase enzyme of the gut microbiota (Wang et al., 2011[28]; Ufnal et al., 2015[27]). TMA is converted to TMAO by the FMO3 enzyme in the liver and excreted by the kidneys (Al-Waiz et al., 1987[1]; Tomlinson and Wheeler, 2017[26]). In recent years, increased TMAO levels have been associated with cardiovascular adverse events' risk (Xie et al., 2021[29]; Yang et al., 2021[30]; Baranyi et al., 2022[3]; Chen et al., 2022[6]). Studies also showed that TMAO might be associated with diabetes, cancer, and inflammatory and neurological diseases (Cho and Caudill, 2017[7]; Chan et al., 2019[5]; Brunt et al., 2021[4]; Gatarek and Kaluzna-Czaplinska, 2021[15]). In conclusion, it is suggested that serum/urine TMAO levels may be a biomarker for the prognosis of diseases and, the determination of serum/urine TMAO levels in humans became crucial. 

Chromatographic analysis and mass detection methods of biomolecules stand as benchmark techniques for diagnosis and treatment. Likewise, serum and plasma TMAO levels have been measured by several methods, including chemical method (El‐Deeb et al., 2019[10]), nuclear magnetic resonance (NMR) (Sanchez-Alcoholado et al., 2017[24]; Garcia et al., 2019[13]), high performance liquid chromatography (HPLC) (Gu et al., 2020[17]), liquid chromatography‐mass spectrometry (LC-MS) (Eyupoglu et al., 2019[12]), liquid chromatography-tandem mass spectrometry (LC-MS/MS) (Colaco et al., 2021[8]) and high performance liquid chromatography-tandem mass spectrometry (HPLC-MS/MS) (Enko et al., 2020[11]). Some studies used different measurement techniques for the TMAO levels depending on *in vitro* or *in vivo* experimental design (Gautam et al., 2019[16]), and some compared the reliability of different quantification methods NMR vs. MS (Garcia et al., 2017[14]). However, due to high costs and specialization necessity, the application of these methods is not common in clinical laboratories. 

Micro-enzyme-linked immunosorbent assay (ELISA) is another standard technique for determining biomolecules, such as TMAO (Liu et al., 2022[20]; Ozorowski et al., 2022[23]) and most clinical laboratories have ELISA systems. ELISA technique is based on antigen-antibody coupling and enzymatic reaction, which detects the target protein in the samples. ELISA is an appropriate and commonly used method for small-scale scientific research. There are several advantages and disadvantages of this technique. ELISA is a simple, rapid, practical, cost-effective procedure with high sensitivity and specificity. Besides these advantages, standardization/validation of the ELISA kit is essential to use as a diagnostic technique due to stability and reagent cross-reactivity problems in the clinical setting (Tighe et al., 2015[25]; Hosseini et al., 2018[18]). Moreover, the ELISA protocols used for detecting the same parameter may differ according to the companies and standardization of these protocols is suggested according to the laboratory conditions (Correa et al., 2021[9]). 

Most studies have used HPLC or LC-MS/MS techniques to detect TMAO levels, we chose more applicable ELISA method to determine serum TMAO levels in another project. However, we had problems obtaining accurate standard curves with the original protocol and decided to modify this protocol. Low transparency due to poor reporting of methodology is an important factor affecting the reproducibility of studies (Klein, 2022[19]). Hence, we believe in reporting and sharing every step-in laboratory research, especially the methodology. Reporting this optimized ELISA protocol, we aimed to reach researchers to prevent from failing time and funds.

## Materials and Methods

Human TMAO ELISA kits (E4733Hu) were obtained from Bioassay Technology (BT) Laboratory (China) and stored at -20 °C until the day of the experiment. The kits with the same lot number (202101016) were used for all experiments. Two ELISA kits were applied with original kit protocol and five ELISA kits were with modified kit protocol. This kit is a sandwich-type ELISA developed to quantitatively determine TMAO levels in human serum, plasma, cell culture supernatants, cell lysates, and tissue homogenates. In this study, only the part of the protocol related to the standard solutions was included, and the part related to samples was not described. Before starting the experiment, the protocol and expiration date of the commercial kits were checked, and kit solutions reached room temperature. The calibrated automatic micropipettes were used, and the pipette tip was changed every step during the experiments. All experiments were performed by the same person with the same devices under the same conditions. Reagent and resource information and related links were given in Table 1(Tab. 1). 

### Preparation of standard dilutions

Standard and standard diluent solutions were gently shaken before dilution. 32 ng/ml standard solution was obtained by taking 120 μl of 64 ng/ml standard stock solution and was diluted with 120 μl standard diluent. This process was repeated to obtain standards at 16 ng/ml, 8 ng/ml, 4 ng/ml, and 2 ng/ml concentrations, respectively.

### Preparation of wash buffer

The kit contains 20 ml concentrated wash buffer (25x). Distilled water was added onto concentrated wash buffer (20 ml) until reaching 500 ml volume to obtain 1x wash buffer. Wash buffer was freshly prepared on the experiment day.

### BT Human TMAO ELISA kit protocol

**Figure 1:**
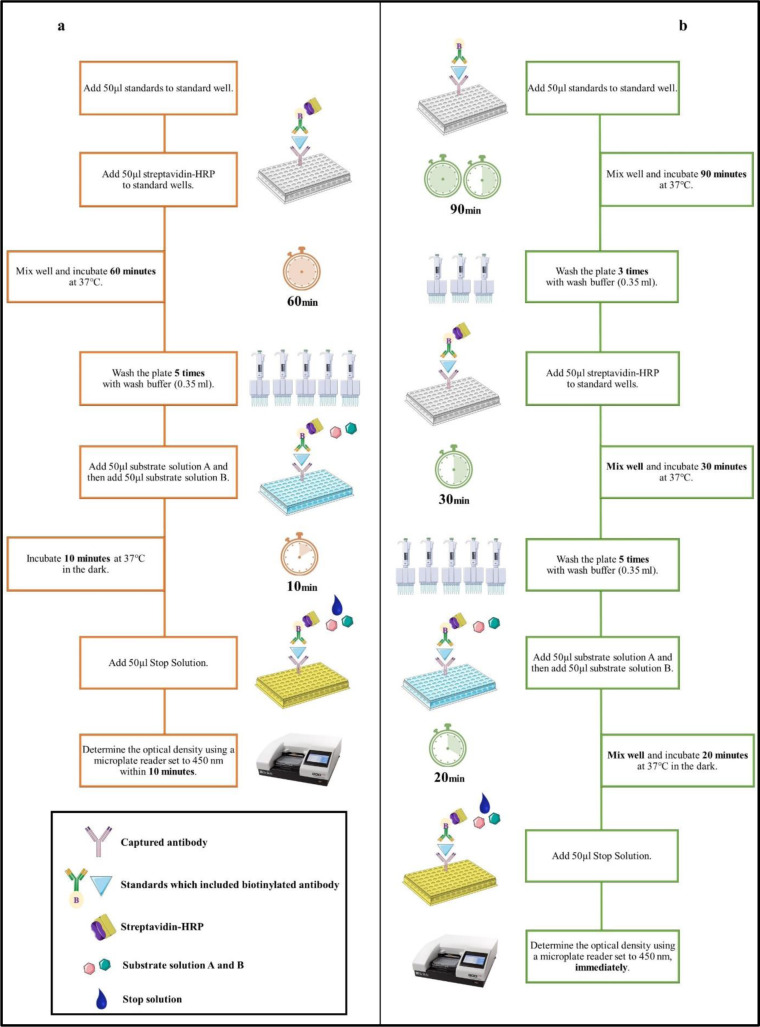
Flowchart of original BT human TMAO ELISA kit protocol (a) and modified BT Human TMAO ELISA kit protocol (b). The figure has been prepared using the Servier Medical Art website (https://smart.servier.com/).

The commercial kit protocol is summarized in Figure 1a(Fig. 1). 64 ng/ml, 32 ng/ml, 16 ng/ml, 8 ng/ml, 4 ng/ml and 2 ng/ml standard solutions (50 μl) were added in duplicate to the standard wells vertically. Since the standard solution in the kit contains biotinylated antibody, the antibody was not added to the standard wells. Two wells were spared empty as blank controls. Then, 50 μl streptavidin-horse radish peroxidase (HRP) was added to the standard wells, while not into the blank control wells. The plate was covered with a sealer, mixed on the shaker for 2-3 minutes, and then placed in the incubator at 37 °C for 60 minutes. After the incubation period, the solutions in the plate were poured out. 350 µl washing buffer was filled into the wells using the multichannel pipette manually and poured after waiting for 1 minute, and this process was repeated five times. The wash solution in the wells was removed from the plate using filter paper and paper towels. First, 50 μl substrate solution A was added to all wells, including the blank control. Then, 50 μl substrate solution B was applied, protected from light. The plate was covered with a sealer and incubated at 37 °C for 10 minutes. Following the incubation, 50 μl stop solution was added to all wells, and the blue color instantly turned yellow. The optical density was determined with a microplate reader immediately (BioTek ELX800, USA) at 450 nm. 

### Modified BT Human TMAO ELISA kit protocol

The modified protocol is summarized in Figure 1b(Fig. 1). The standard dilutions (32 ng/ml, 16 ng/ml, 8 ng/ml, 4 ng/ml and 2 ng/ml) were prepared as described above the original kit protocol. 50 µl of standard solutions (64 ng/ml, 32 ng/ml, 16 ng/ml, 8 ng/ml, 4 ng/ml and 2 ng/ml) were applied in duplicate. The plate was covered with a sealer, mixed on the shaker for 2-3 minutes, and then incubated at 37 °C for 90 minutes. After the incubation, the solutions in the plate were poured out. Then, 350 µl of washing buffer was filled into the wells and poured out after waiting for 1 minute, and this process was repeated three times. The plate was dried on filter paper and paper towels. Then, 50 μl of streptavidin-HRP was applied to the standard wells, while not into the blank control wells. The plate was covered with a sealer and mixed on the shaker for 2-3 minutes, then incubated at 37 °C for 30 minutes. After the incubation, the washing process of the plate was repeated five times and dried as described above. Then, 50 µl of substrate solution A and 50 µl of substrate solution B, protected from light, were added to all wells, respectively. The plate was sealed, mixed on the shaker for 2-3 minutes, and incubated at 37 °C for 20 minutes. Following, 50 μl of stop solution was applied to all wells, and the blue color instantly turned yellow. The optical density was measured at 450 nm immediately.

### Statistical analysis

Data were presented as mean ± standard deviation. Standard concentrations were run in duplicate throughout all experiments. Concentration absorbance curves were plotted by using mean values for each ELISA kit. Each individual standard concentration absorbance data is considered as a technical replicate to compare the effect of both kit protocols on the deviation of standard concentrations. Data fitting a normal distribution was assessed by using the Shapiro-Wilk test. Concentration difference at each concentration was evaluated by using Student's t test and p < 0.05 was considered as statistically significant. All graphs have been prepared using the GraphPad Prism 9.5.0 (USA).

## Results

### Standard curve of original BT human TMAO ELISA kit protocol

**Figure 2:**
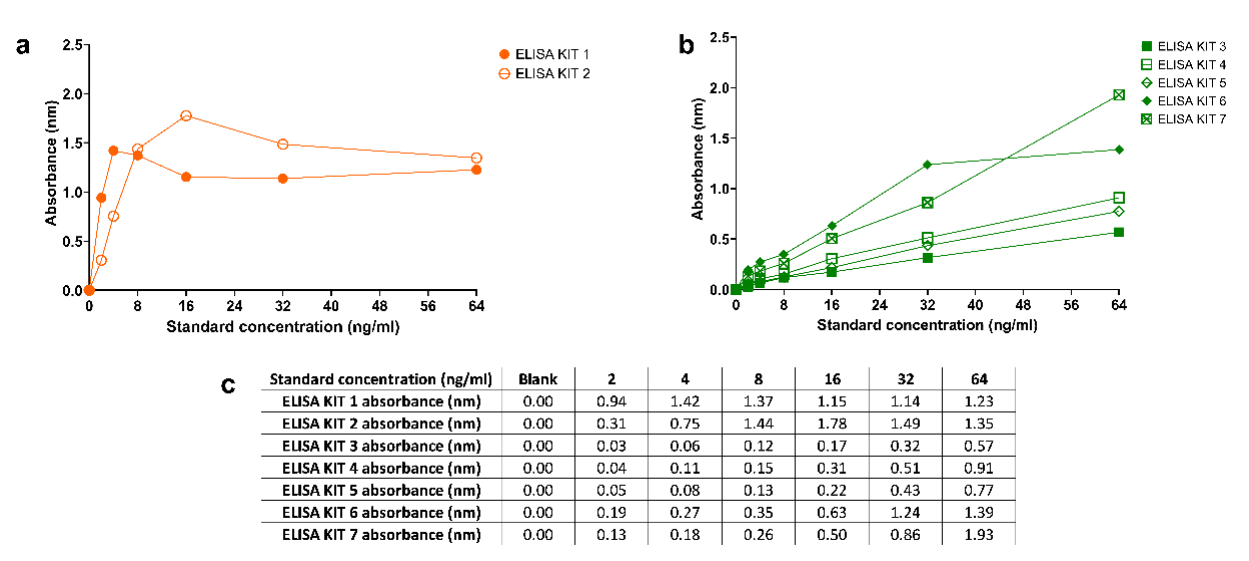
a. Graphs of absorbance values for the original BT Human TMAO ELISA kit protocol. b. Graphs of absorbance values for the modified BT Human TMAO ELISA kit protocol. c. Mean absorbance values corresponding to each standard concentration.

After performing the original protocol step by step, we detected that the blue color changed to yellow in the standard wells, and we determined the absorbance of the standard solution. However, we found that the absorbances of standard solutions were inconsistent with their concentrations in ELISA KIT 1 (Figure 2c(Fig. 2)). Although the concentration of the standard solution decreased, we measured higher absorbance, and the accurate standard curve was not obtained. Similar results were obtained in ELISA KIT 2 (Figure 2c(Fig. 2)). Graphics obtained from these experiments were given in Figure 2a(Fig. 2). First, we evaluated the troubleshooting suggestions of the kit. They were about the high background, weak/no signal, and low sensitivity, and these solutions did not help to overcome the existing problems in our study. Therefore, we decided to modify the original kit protocol by utilizing protocols of other human TMAO ELISA kits on the market. 

### Evaluation of different human TMAO ELISA kit protocols

Human TMAO ELISA kits from SunRed Biotechnology Company, MyBioSource, and ELK Biotechnology companies on the market were compared to BT Laboratory (Table 2(Tab. 2) and Supplementary Information). The protocol of SunRed Biotechnology Company and MyBioSource was found to overlap with BT Laboratory protocol. When the differences in these three kits were examined, the BT Laboratory and MyBioSource kits contain substrate A and B as the substrate solution, while the SunRed Biotechnology Company kit has chromogen solution A and B. Also, a longer incubation time (15-20 minutes) with substrate solutions is recommended in the MyBioSource protocol. 

ELK Biotechnology kit protocol differs significantly from other kits. This kit has the most prolonged protocol duration. In other kits, standards and samples are incubated with biotinylated antibodies and streptavidin-HRP concomitantly for 60 minutes. However, in ELK Biotechnology protocol, only standards and samples are incubated for 80 minutes without adding biotinylated antibodies and streptavidin-HRP. In addition, ELK Biotechnology kit does not contain biotinylated antibodies in standard solutions, and biotinylated antibodies must be added to each well and incubated for 50 minutes. The incubation period with streptavidin-HRP is 50 minutes. Washing step between each incubation is recommended in this kit (3-3-5 times, respectively). This kit contains 3,3',5,5'-Tetramethylbenzidine (TMB) as the substrate solution, and the incubation time is 20 minutes.

Optical density measuring is recommended at 450 nm in all kits. Immediately reading for MyBioSource and ELK Biotechnology kits, while reading within 10 and 15 minutes for BT Laboratory and SunRed Biotechnology Company kits are recommended, respectively.

### Standard curve of modified BT Human TMAO ELISA kit protocol

Three different kits have similar protocols which we could not obtain trustworthy standard absorbances. For this reason, we modified our protocol based on the ELK Biotechnology protocol. However, we changed some steps because the standard solution does not contain biotinylated antibodies in this kit. When deciding on these modifications, we benefited from other human ELISA protocols.

As a result, after applying the modified protocol, the absorbances of standard solutions were found to be consistent with their concentrations in ELISA KIT 3, 4, 5, 6, 7 (Figure 2c(Fig. 2)). Lower absorbance values were measured at lower concentrations of standard solutions as expected. The absorbance values and graphics obtained from these experiments were given in Figure 2b(Fig. 2). 

**Figure 3:**
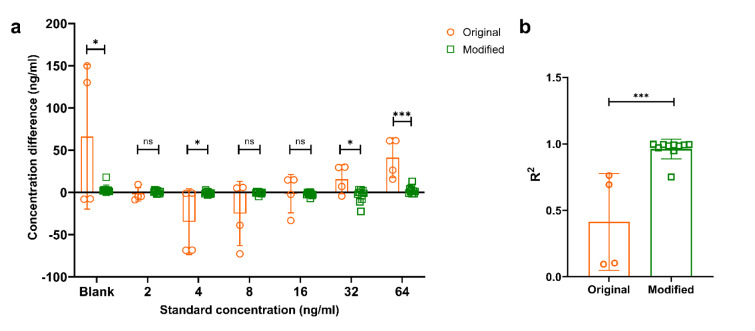
a. Concentration difference of standard solutions at each point; b. R^2^ values. Values are given as mean ± standard deviation. Student's t-test was used for statistical analysis. n=4, original kit protocol; n=10, modified kit protocol. ns, non-significant; *p < 0.05, ***p<0.001

The absolute differences in standard concentrations were found significantly higher at 64 ng/ml, 32 ng/ml, 4 ng/ml and at the blank points in the original kit protocol compared to modified kit protocol (Figure 3a(Fig. 3)). At other concentration points, there was a marked difference in original kit protocol; however, the results were not statistically significant due to high standard deviation and small sample size. In ELISA experiments, R^2 ^value reflects a good standard curve. As this R^2 ^value closes to 1, it shows the accuracy of the experiments. The mean R^2 ^value was found to be closer to 1 when modified kit protocol was applied (R^2^, 0.41 ± 0.36; 0.96 ± 0.08 in original kit protocol and modified kit protocol, respectively) (Figure 3b(Fig. 3)).

## Discussion

In our study, we optimized the commercially available human TMAO ELISA kit to obtain trustworthy data. Because when we applied the original ELISA kit protocol, we obtained higher absorbance values in the wells with lower standard solution concentrations. Higher R^2 ^values and lower deviation of standard concentrations in modified kit protocol show that the modified human TMAO ELISA kit protocol will provide more reliable results. 

Prior to modifying the protocol, we evaluated the potential problems with the kit. We ruled out the possibility of captured antibody and biotinylated antibody absence since we could measure the absorbance in standard wells. Thus, it was assumed the plate was coated with captured antibody and standard solutions contain biotinylated antibody. We detected yellow color which shows the occurrence of an enzymatic reaction between streptavidin-HRP and substrate solutions. So, we accepted that these molecules are functional and not degraded. 

An unsuccessful standard curve in ELISA assay makes interpretation of the data difficult and may result in time, workforce, and money loss. Researchers proposed to generate a new standard curve from sample ΔOD values to predict sample concentrations (Natarajan and Remick, 2008[22]) which may not be applicable to each ELISA assay. Moreover, even the assumption of extreme sample absorbance values by using curve expect programs is not recommended despite the successful standard curve (Aydin, 2015[2]). Therefore, we decided to make modifications according to the several commercially available ELISA kit protocols to improve the procedure. 

We examined different protocols from several biotechnology companies and noticed that the incubation time was longer to enhance binding of molecules. We increased the first incubation period, which may augment the binding of TMAO to the coated antibody on the plate. In the original protocol, the manufacturer suggests adding standard solution which contained biotinylated antibody and HRP-streptavidin concomitantly. However, it may lead binding of HRP-streptavidin to free TMAO molecules before saturation of TMAO with coated antibody. Additionally, we realized many kits suggest adding a washing process between steps. Proper washing prevents unbound molecules effect on the results (Hosseini et al., 2018[18]). Therefore, we added a washing step before incubation of HRP-streptavidin to facilitate the removal of free TMAO molecules. In this way, we may prevent non-specific binding and get more accurate results. 30 minutes incubation with HRP-streptavidin was enough for our study to bind HRP-streptavidin to biotinylated antibody. Last, after the washing period, we incubated substrate solutions for 20 minutes instead of 10 minutes. Prolonged incubation time may strengthen the reaction between HRP and substrate solutions. More washing steps and prolonged incubation time lead to extended experimental period. Researchers may prefer short protocols to save time; however, based on our experience, shorter ELISA protocols may mislead data. 

## Conclusion

In this article, a modified protocol for the human TMAO ELISA method is presented. The modified human TMAO ELISA kit protocol will enable the detection of TMAO in laboratories without the need for expensive devices, prevent the loss of resources in laboratories that will conduct research in this field, and enable researchers to use their time more effectively. 

## Declaration

### Declaration of competing interest

The authors declare that they have no conflict of interest.

### Authors' contributions

Zinnet Sevval Aksoyalp: Conceptualization, funding acquisition, validation, formal analysis, visualization, writing - original draft, review & editing; Betül Rabia Erdogan: Conceptualization, investigation, validation, writing - original draft, review & editing; Saliha Aksun: Conceptualization, validation**, **supervision, writing - original draft, review & editing.

### Acknowledgment

We wish to thank Bioassay Technology Laboratory (China). We contacted the company through the local distributor when we could not obtain accurate curves in two ELISA kits. After filling out the problems of ELISA kits form, they provided us with two replacement kits. 

### Funding

This study was supported by Izmir Katip Celebi University Scientific Research Projects Coordination Unit (Grant number 2020-COV-ECZF-0004). 

## Supplementary Material

Supplementary information

## Figures and Tables

**Table 1:**
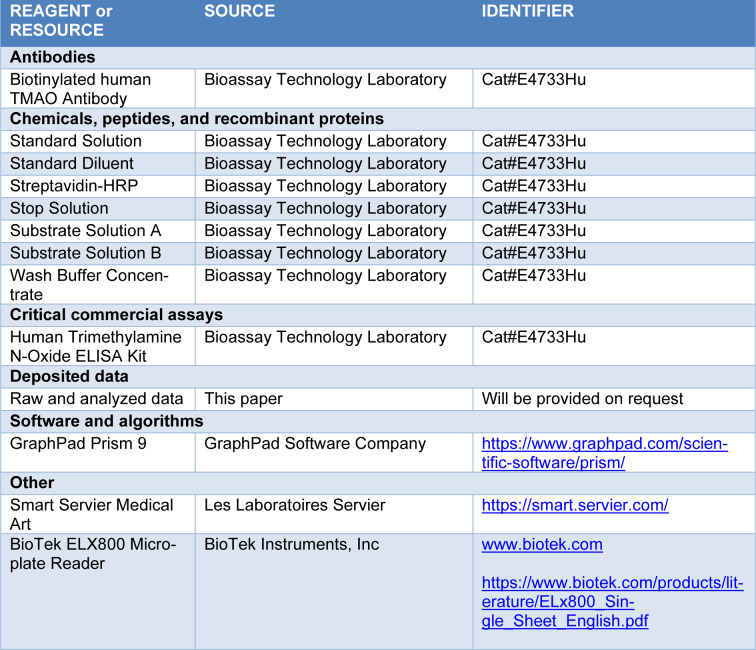
STAR METHOD key sources table

**Table 2:**
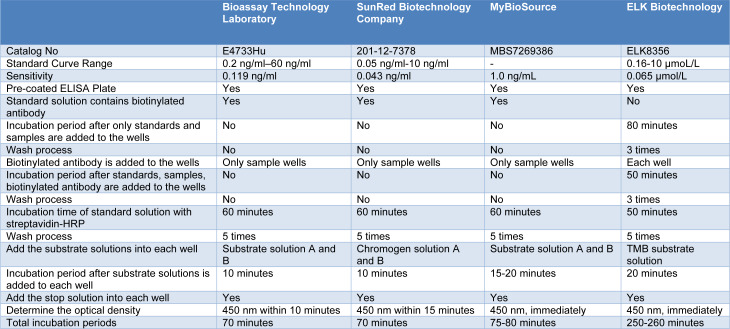
Comparison of different commercial human TMAO ELISA kit protocols
