# Supplementary material for: Optimization of enzyme-linked immunosorbent assay kit protocol to detect trimethylamine N-oxide levels in humans
Source: EXCLI J. 2023 Feb 20;22:263–73. doi: 10.17179/excli2022-5617 (PMC10415589; doi:10.17179/excli2022-5617)
Supplement: Supplementary information [file EXCLI-22-263-s-001.pdf]

EXCLI Journal 2023;22:263-273 – ISSN 1611-2156
Received: November 18, 2022, accepted: February 14, 2023, published: February 20, 2023

Supplementary information to:

Methods:

OPTIMIZATION OF ENZYME-LINKED IMMUNOSORBENT ASSAY KIT PROTOCOL TO DETECT TRIMETHYLAMINE N-OXIDE LEVELS IN HUMANS

Zinnet Sevval Aksoyalp[1], Betül Rabia Erdoğan[1*], Saliha Aksun[2]

[1] Izmir Katip Celebi University, Faculty of Pharmacy, Department of Pharmacology, Izmir, TR
[2] Izmir Katip Celebi University, Faculty of Medicine, Department of Medical Biochemistry, Izmir, TR

* **Corresponding author:** Betül Rabia Erdoğan, Izmir Katip Celebi University, Faculty of Pharmacy, Department of Pharmacology, Izmir, TR.
E-mail: betulrabia.erdogan@ikcu.edu.tr

https://dx.doi.org/10.17179/excli2022-5617

This is an Open Access article distributed under the terms of the Creative Commons Attribution License (http://creativecommons.org/licenses/by/4.0/).

Bioassay Technology Laboratory

Optimize Your Research

Human Trimethylamine-N-Oxide ELISA Kit

USER INSTRUCTION

Cat.No E4733Hu

Standard Curve Range: 0.2ng/ml - 60ng/ml

Sensitivity: 0.119ng/ml

Size: 96 wells

Storage: Store the reagents at 2-8°C. For over 6-month storage refer to the expiration date keep it at -20°C. Avoid repeated thaw cycles. If individual reagents are opened it is recommended that the kit be used within 1 month.

***This product is for research use only, not for use in diagnosis procedures. It's highly recommend to read this instruction entirely before use.**

Precision

Intra-Assay Precision (Precision within an assay) Three samples of known concentration were tested on one plate to assess intra-assay precision.

Inter-Assay Precision (Precision between assays) Three samples of known concentration were tested in separate assays to assess inter-assay precision.

CV(%) = SD/mean x 100

Intra-Assay: CV<8%

Inter-Assay: CV<10%

Intended Use

This sandwich kit is for the accurate quantitative detection of human Trimethylamine-N-Oxide (also known as TMAO) in serum, plasma, cell culture supernates, cell lysates, tissue homogenates.

Assay Principle

This kit is an Enzyme-Linked Immunosorbent Assay (ELISA). The plate has been pre-coated with human TMAO antibody. TMAO present in the sample is added and binds to antibodies coated on the wells. And then biotinylated human TMAO Antibody is added and binds to TMAO in the sample. Then Streptavidin-HRP is added and binds to the Biotinylated TMAO antibody. After

incubation unbound Streptavidin-HRP is washed away during a washing step. Substrate solution is then added and color develops in proportion to the amount of human TMAO. The reaction is terminated by addition of acidic stop solution and absorbance is measured at 450 nm.

Reagent Provided

Components	Quantity
Standard Solution (64ng/ml)	0.5ml x1
Pre-coated ELISA Plate	12 * 8 well strips x1
Standard Diluent	3ml x1
Streptavidin-HRP	6ml x1
Stop Solution	6ml x1
Substrate Solution A	6ml x1
Substrate Solution B	6ml x1
Wash Buffer Concentrate (25x)	20ml x1
Biotinylated human TMAO Antibody	1ml x1
User Instruction	1
Plate Sealer	2 pics
Zipper bag	1 pic

Material Required But Not Supplied

- 37°C±0.5°C incubator
- Absorbent paper
- Precision pipettes and disposable pipette tips
- Clean tubes
- Deionized or distilled water
- Microplate reader with 450 ± 10nm wavelength filter

Precautions

- Prior to use, the kit and sample should be warmed naturally to room temperature 30 minutes.
- This instruction must be strictly followed in the experiment.
- Once the desired number of strips has been removed, immediately reseal the bag to protect the remain from deterioration. Cover all reagents when not in use.
- Make sure pipetting order and rate of addition from well-to-well when pipetting reagents.
- Pipette tips and plate sealer in hand should be clean and disposable to avoid cross-contamination.

- Avoid using the reagents from different batches together.
- Substrate solution B is sensitive to light, don’t expose substrate solution B to light for a long time.
- Stop solution contains acid. Please wear eye, hand and skin protection when using this material. Avoid contact of skin or mucous membranes with kit reagent.
- The kit should not be used beyond the expiration date.

Specimen Collection

Serum Allow serum to clot for 10-20 minutes at room temperature. Centrifuge at 2000-3000 RPM for 20 minutes.

Plasma Collect plasma using EDTA or heparin as an anticoagulant. Centrifuge samples for 15 minutes at 2000-3000 RPM at 2 - 8°C within 30 minutes of collection.

Urine Collect by sterile tube. Centrifuge at 2000-3000 RPM for approximately 20 minutes. When collecting pleuroperitoneal fluid and cerebrospinal fluid, please follow the procedures above-mentioned.

Cell Culture Supernatant Collect by sterile tubes when examining secrete components. Centrifuge at 2000-3000 RPM for approximately 20 minutes. Collect the supernatants carefully. When examining the components within the cell, use PBS (pH 7.2-7.4) to dilute cell suspension to the cell concentration of approximately 1 million/ml. Damage cells through repeated freeze-thaw cycles to let out the inside components. Centrifuge at 2000-3000 RPM for approximately 20 minutes.

Tissue and other body fluids Rinse tissues in PBS (pH 7.4) to remove excess blood thoroughly and weigh before homogenization. Mince tissues and homogenize them in PBS (pH7.4) with a glass homogenizer on ice. Thaw at 2-8°C or freeze at -20°C. Centrifuge at 2000-3000 RPM for approximately 20 minutes.

Note

- Sample concentrations should be predicted before being used in the assay. If the sample concentration is not within the range of the standard curve, users must **contact us** to determine the optimal sample for their particular experiments.
- Samples to be used within 5 days should be stored at 2-8°C. Samples should be aliquoted or must be stored at -20°C within 1 month or -80°C within 6 months. Avoid repeated freeze thaw cycles.
- Samples should be brought to room temperature before starting the assay.

- Centrifuge to collect sample before use.
- Samples containing NaN3 can't be tested as it inhibits the activity of Horse Radish Peroxidase (HRP).
- Collect the supernatants carefully. When sediments occurred during storage, centrifugation should be performed again.
- Hemolysis can greatly impact the validity of test results. Take care to minimize hemolysis.

Sample can't be diluted with this kit. Owing to the the material we use to prepare the kit, the sample matrix interference may falsely depress the specificity and accuracy of the assay.

Reagent Preparation

- All reagents should be brought to room temperature before use.
- **Standard** Reconstitute the 120μl of the standard (64ng/ml) with 120μl of standard diluent to generate a 32ng/ml standard stock solution. Allow the standard to sit for 15 mins with gentle agitation prior to making dilutions. Prepare duplicate standard points by serially diluting the standard stock solution (32ng/ml) 1:2 with standard diluent to produce 16ng/ml, 8ng/ml, 4ng/ml and 2ng/ml solutions. Standard diluent serves as the zero standard(0 ng/ml). Any remaining solution should be frozen at -20°C and used within one month. Dilution of standard solutions suggested are as follows:

32ng/ml	Standard No.5	120μl Original Standard + 120μl Standard Diluent
16ng/ml	Standard No.4	120μl Standard No.5 + 120μl Standard Diluent
8ng/ml	Standard No.3	120μl Standard No.4 + 120μl Standard Diluent
4ng/ml	Standard No.2	120μl Standard No.3 + 120μl Standard Diluent
2ng/ml	Standard No.1	120μl Standard No.2 + 120μl Standard Diluent

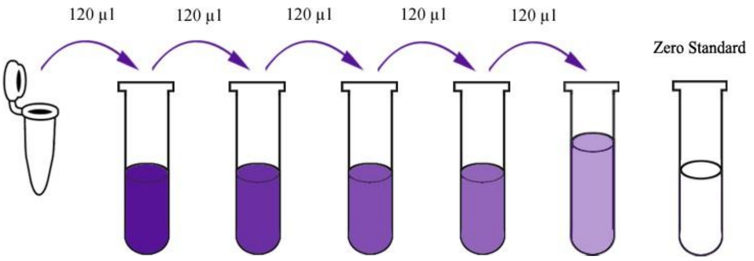

Standard Concentration	Standard No.5	Standard No.4	Standard No.3	Standard No.2	Standard No.1
64ng/ml	32ng/ml	16ng/ml	8ng/ml	4ng/ml	2ng/ml

- **Wash Buffer** Dilute 20ml of Wash Buffer Concentrate 25x into deionized or distilled water to yield 500 ml of 1x Wash Buffer. If crystals have formed in the concentrate, mix gently

until the crystals have completely dissolved.

Assay Procedure

1. Prepare all reagents, standard solutions and samples as instructed. Bring all reagents to room temperature before use. The assay is performed at room temperature.
2. Determine the number of strips required for the assay. Insert the strips in the frames for use. The unused strips should be stored at 2-8°C.
3. Add 50μl standard to standard well. **Note**: Don't add antibody to standard well because the standard solution contains biotinylated antibody.
4. Add 40μl sample to sample wells and then add 10μl anti-TMAO antibody to sample wells, then add 50μl streptavidin-HRP to sample wells and standard wells (Not blank control well). Mix well. Cover the plate with a sealer. Incubate 60 minutes at 37°C.
5. Remove the sealer and wash the plate 5 times with wash buffer. Soak wells with at least 0.35 ml wash buffer for 30 seconds to 1 minute for each wash. For automated washing, aspirate all wells and wash 5 times with wash buffer, overfilling wells with wash buffer. Blot the plate onto paper towels or other absorbent material.
6. Add 50μl substrate solution A to each well and then add 50μl substrate solution B to each well. Incubate plate covered with a new sealer for 10 minutes at 37°C in the dark.
7. Add 50μl Stop Solution to each well, the blue color will change into yellow immediately.
8. Determine the optical density (OD value) of each well immediately using a microplate reader set to 450 nm within 10 minuets after adding the stop solution.

Summary

1. Prepare all reagents, samples and standards.
2. Add sample and ELISA reagent into each well. Incubate for 1 hour at 37°C.
3. Wash the plate 5 times.
4. Add substrate solution A and B. Incubate for 10 minutes at 37°C.
5. Add stop solution and color develops.
6. Read the OD value within 10 minutes.

Calculation of Result

Construct a standard curve by plotting the average OD for each standard on the vertical (Y) axis against the concentration on the horizontal (X) axis and draw a best fit curve through the points on the graph. These calculations can be best performed with computer-based curve-fitting software and the best fit line can be determined by regression analysis.

Typical Data

This standard curve is only for demonstration purposes. A standard curve should be generated with each assay.

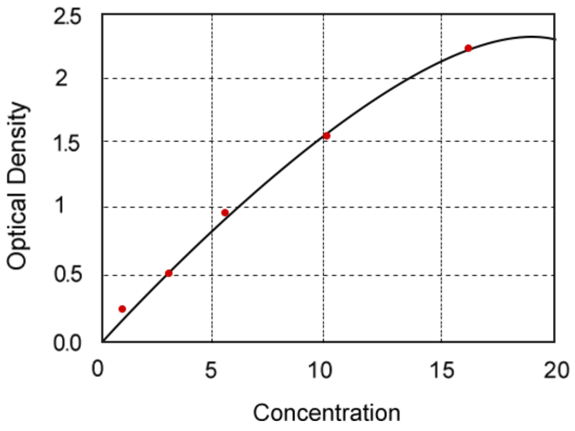

Troubleshooting

Possible Case	Solution
**High Background**	
• Improper washing	• Increasing duration of soaking steps
• Substrate was contaminated	• Replace. Substrate should be clean and avoid crossed contamination by using the sealer
• Non-specific binding of antibody	• Replace another purified antibody or blocking buffer
• Plate are not be sealing incompletely	• Make sure to follow the instruction strictly
• Incorrect incubation temperature	• Incubate at room temperature
• Substrate exposed to light prior to use	• Keep substrate in a dark place
• Contaminated wash buffer	• Use a clean buffers and sterile filter
**Weak Signal**	
• Improper washing	• Increasing duration of soaking steps
• Incorrect incubation temperature	• Incubate at room temperature
• Antibody are not enough	• Increase the concentration of the antibody
• Reagent are contaminated	• Use new one
• Pipette are not clean	• Pipette should be clean
**No Signal**	
• Reagent are contaminated	• Use new one
• Sample prepared incorrectly	• Make sure the sample workable/dilution
• Antibody are not enough	• Increase the antibody concentration
• Wash buffer contains sodium azide	• Use a new wash buffer and avoid sodium azide in it
• HRP was not added	• Add HRP according to the instruction
**Poor Precision**	
• Imprecise/ inaccurate pipetting	• Check/ calibrate pipettes
• Incomplete washing of the wells	Make sure wells are washed adequately by filling the wells with wash buffer and all residual antibody solutions crossed well before washing.

If you have any question on the order please contact us via: order@bt-laboratory.com;
technical assistance please contact us via: support@bt-laboratory.com
More product visit *www.bt-laboratory.com*

Human（TMAO）
ELISA Kit Instruction

Catalogue No.

201-12-7378

Preface

Please carefully read this instruction before using. This ELISA kit is based on the principle of double-antibody sandwich technique to detect Human (TMAO). Be used only for research purposes, not be used for medical diagnosis.

Full Name

Human Trimethylamine-N-oxide (TMAO) ELISA Kit

Intended Use

This kit is used to assay the Trimethylamine-N-oxide (TMAO) in the sample of human’ s serum, blood plasma, and other related tissue Liquid.

Test principle

The kit uses a double-antibody sandwich enzyme-linked immunosorbent assay (ELISA) to assay the level of Human Trimethylamine-N-oxide (TMAO)in samples. Add Trimethylamine-N-oxide (TMAO)to monoclonal antibody Enzyme well which is pre- coated with Human Trimethylamine-N-oxide (TMAO) monoclonal antibody, incubation; then, add Trimethylamine-N-oxide (TMAO)

EMAIL:shanghai@sunredbio.com WEB:www.srbooo.com
TEL:86-21-51877391 FAX:021-51564103

antibodies labeled with biotin, and combined with Streptavidin-HRP to form immune complex; then carry out incubation and washing again to remove the uncombined enzyme. Then add Chromogen Solution A, B, the color of the liquid changes into the blue, And at the effect of acid, the color finally becomes yellow. The chroma of color and the concentration of the Human Substance Trimethylamine-N-oxide (TMAO) of sample were positively correlated.

Materials supplied in the Test Kit

1	Standard（12.8ng/ml）	0.5ml
2	Standard diluent	3ml
3	Microelisa Strip plate	12well×8strips
4	Str- HRP-Conjugate Reagent	6ml
5	30×wash solution	20ml
6	Biotin- TMAO Ab	1ml
7	Chromogen Solution A	6ml
8	Chromogen Solution B	6ml
9	Stop Solution	6ml
10	Instruction	1
11	Closure plate membrane	2
12	Sealed bags	1

Materials required but not supplied

1. 37 ℃ incubator
2. Standard Enzyme reader

EMAIL:shanghai@sunredbio.com WEB:www.srbooo.com
TEL:86-21-51877391 FAX:021-51564103

3. Precision pipettes and Disposable pipette tips
4. Distilled water
5. Disposable tubes for sample dilution
6. Absorbent paper

Important Notes

1. Been taken out from the 2-8℃ environment, the kit should be balanced 30 minutes in the ambient temperature then use. If the Coated plates of Enzyme haven' t been used up after opened, the remaining plates should be stored in Sealed bag.

2. For each step, add Sample with sample injector which should be calibrated frequently, in order to avoid unnecessary experimental tolerance.

3. he operation shall be carried out accordance to the instructions strictly. And test results must be based on the readings of the Enzyme reader.

4. In order to avoid cross-contamination, it is forbidden to re-use the suction head and seal plate membrane in your hands.

5. All samples, washing buffer and each kind of reject should according to infective material process.

6. The idle agents shall be put up or covered. Do not use reagent with different batches. And use them before expired date.

7. The substrate B is light-sensitive. Prolonged exposure to light is forbidden.

Washing method

Manually washing method: shake away the remain liquid in the enzyme plates;

EMAIL:shanghai@sunredbio.com WEB:www.srbooo.com
TEL:86-21-51877391 FAX:021-51564103

place some bibulous papers on the test-bed, and flap the plates on the upside down strongly. Inject at least 0.35ml after-dilution washing solution into the well, and marinate 1~2 minutes. Repeat this process according to your requirements.

Automatic washing method: if there is automatic washing machine, it should only be used in the test when you are quite familiar with its function and performance.

Specimen requirements

1. Can' t detect the sample which contain NaN3, because NaN3 inhibits HRP active

2. extract as soon as possible after Specimen collection, and according to the relevant literature, and should be experiment as soon as possible after the extraction. If it can' t, specimen can be kept in -20 ℃ to preserve, Avoid repeated freeze-thaw cycles.

3. serum- coagulation at room temperature 10-20 mins, centrifugation 20-min at the speed of 2000-3000 r.p.m. remove supernatant, If precipitation appeared, Centrifugal again.

4. plasma-use suited EDTA or citrate plasma as an anticoagulant, mix 10-20 mins ,centrifugation 20-min at the speed of 2000-3000 r.p.m. remove supernatant, If precipitation appeared, Centrifugal again.

5. Urine-collect sue a sterile container, centrifugation 20-min at the speed of 2000-3000 r.p.m. remove supernatant, If precipitation appeared, Centrifugal again. The Operation of Hydrothorax and cerebrospinal fluid Reference to it.

6. cell culture supernatant-detect secretory components, collect sue a

EMAIL:shanghai@sunredbio.com WEB:www.srbooo.com
TEL:86-21-51877391 FAX:021-51564103

sterile container, centrifugation 20-min at the speed of 2000-3000 r.p.m. remove supernatant,detect the composition of cells, Dilute cell suspension with PBS（PH7.2-7.4）, Cell concentration reached 1 million / ml, repeated freeze-thaw cycles, damage cells and release of intracellular components, centrifugation 20-min at the speed of 2000-3000 r.p.m. remove supernatant, If precipitation appeared, Centrifugal again.

7. Tissue samples- After cutting samples, check the weight,add PBS（PH7.2-7.4）, Rapidly frozen with liquid nitrogen, maintain samples at 2-8℃ after melting,add PBS（PH7.4）, Homogenized by hand or Grinders, centrifugation 20-min at the speed of 2000-3000 r.p.m. remove supernatant.

Note: Grossly hemolyzed samples are not suitable for use in this assay.

Assay procedure

1. Standard dilution:

this test kit will supply one original Standard reagent, please dilute it by yourself according to the instruction.

**6.4ng/ml**	Standard No.5	120μl Original Standard + 120μl Standard diluents
**3.2ng/ml**	Standard No.4	120μl Standard No.5 + 120μl Standard diluents
**1.6ng/ml**	Standard No.3	120μl Standard No.4 + 120μl Standard diluent
**0.8ng/ml**	Standard No.2	120μl Standard No.3 + 120μl Standard diluent
**0.4ng/ml**	Standard No.1	120μl Standard No.2 + 120μl Standard diluent

2. The quantity of the plates depends on the quantities of to-be-tested samples and the standards. It is suggested to duplicate each standard and blank well. Every sample shall be made according to your required quantity, and try to use the duplicated well as possible.

3. Inject samples:

EMAIL:shanghai@sunredbio.com WEB:www.srbooo.com
TEL:86-21-51877391 FAX:021-51564103

① Blank well: don' t add samples and TMAO-antibody labeled with biotin, Streptavidin-HRP, only Chromogen solution A and B, and stop solution are allowed; other operations are the same.

② Standard wells: add standard 50μl, Streptavidin-HRP 50μl(since the standard already has combined biotin antibody, it is not necessary to add the antibody);

③ To be test wells: add sample 40μl, and then add both TMAO-antibody 10μl and Streptavidin-HRP 50μl. Then seal the_sealing memberance, and gently shaking, incubated 60 minutes at 37 ℃.

4. Confection: dilute 30 times the 30×washing concentrate with distilled water as standby.

5. Washing: remove the memberance carefully, and drain the liquid, shake away the remaining water.

6. Add chromogen solution A 50μl, then chromogen solution B 50μl to each well. Gently mixed, incubate for 10 min at 37℃ away from light.

7. Stop: Add Stop Solution 50μl into each well to stop the reaction(the blue changes into yellow immediately).

8. Final measurement: Take blank well as zero , measure the optical densit (OD) under 450 nm wavelength which should be carried out within 15min after adding the stop solution.

9. According to standards' concentration and the corresponding OD values, calculate out the standard curve linear regression equation, and then apply the OD values of the sample on the regression equation to calculate the corresponding sample' s concentration. It is acceptable to use kinds of software to make calculations.

EMAIL:shanghai@sunredbio.com WEB:www.srbooo.com
TEL:86-21-51877391 FAX:021-51564103

Summary procedures

Preparing reagents, samples and standards

⇩

Add prepared samples and standards, antibodies labeled with enzyme, reacting 60 minutes at 37 ℃

⇩

Plate washed five times, adding Chromogen solution A, B, reacting 10 minutes at 37℃

⇩

Add stop solution

⇩

measure the OD value within 10min

⇩

Calculation

Calculate

Take the standard density as the horizontal, the OD value for the vertical, draw the standard curve on graph paper, Find out the corresponding density according to the sample OD value by the Sample curve (the result is the sample density) or calculate the straight line regression equation of the standard curve with the standard density and the OD value ,with the sample OD value in the equation, calculate the sample density.

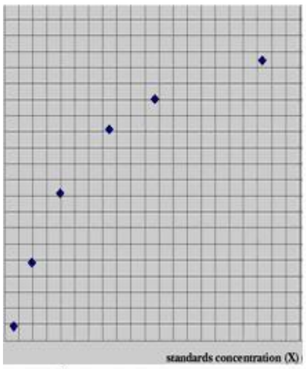

sunred company
provides this chartis for reference only

EMAIL:shanghai@sunredbio.com WEB:www.srbooo.com
TEL:86-21-51877391 FAX:021-51564103

Sensitivity、Assay range

Sensitivity:**0.043ng/ml**

(The sensitivity of this assay was defined as the lowest protein concentration that could be differentiated from zero.It was determined by sub-tracing two standard deviations to the mean optical density value of twenty zero standard replicates and calculating the corresponding concentration.)

Assay range: **0.05ng/ml→10ng/ml**

Intra-assay Precision: 3 samples with low, middle and high level Human TMAO were tested 20 times on one plate, respectively.

Inter-assay Precision: 3 samples with low, middle and high level Human TMAO were tested on 3 different plates, 8 replicates in each plate.

CV(%) = SD/meanX100

Intra-Assay: CV<10%

Inter-Assay: CV<12%

Package size

96T per box

Validity & Storage

six months (2-8℃)

EMAIL:shanghai@sunredbio.com WEB:www.srbooo.com
TEL:86-21-51877391 FAX:021-51564103

Human Trimethylamine-N-Oxide (TMAO)

ELISA Kit (Competitive ELISA)

96 Tests

Catalog Number: MBS7269386

Store all reagents at 2-8°C

Valid Period: six months

For samples:
Serum, plasma, cell culture supernatants, body fluid and tissue homogenate

MyBioSource.com

Important Note!

Sample Preparation:

With respect to 6.2, we suggest pre-experimenting with neat (undiluted) samples, 1:2 or 1:4 dilutions. Please avoid diluting your samples more than 1:10 as it would exceed the dilution limit set for this kit. If the expected concentration of the target is beyond the detection range of the kit, please contact technical support.

* FOR RESEARCH PURPOSES ONLY. NOT FOR USE IN-DIAGNOSTIC PROCEDURES *

Table of Contents

1. INTENDED USE....3
2. PRINCIPLE OF THE ASSAY....3
3. MATERIALS....4
4. SPECIMEN COLLECTION AND STORAGE....5
5. MATERIALS AND EQUIPMENT REQUIRED BUT NOT SUPPLIED....7
6. SAMPLE PREPARATION....7
7. REAGENTS PREPARATION....8
8. ASSAY PROCEDURE....9
9. CALCULATION OF RESULTS....10
10. CERTIFICATE OF ANALYSIS....12
11. SAFETY NOTES....13
12. QUALITY CONTROL....14

12) Incomplete washing will adversely affect the test outcome. All washing must be performed with Wash Solution provided. All residual wash liquid must be drained from the wells by efficient aspiration or by decantation followed by tapping the plate forcefully on absorbent paper. Never insert absorbent paper directly into the wells.

13) Because TMB is light sensitive, avoid prolonged exposure to light. Also avoid contact between TMB and metal, otherwise color may develop.

MyBioSource.com

12. QUALITY CONTROL

1) It is recommended that all standards, controls and samples be run in duplicate. Standards and samples must be assayed at the same time.
2) The coefficient of determination of the standard curve should be ≥ 0.95.
3) Cover or cap all kit components and store at 2-8° C when not in use.
4) Microtiter plates should be allowed to come to room temperature before opening the foil bags. Once the desired number of strips has been removed, immediately reseal the bag with desiccants and store at 2-8°C to maintain plate integrity.
5) Samples should be collected in pyrogen/endotoxin-free tubes.
6) Samples should be frozen if not analyzed shortly after collection. Avoid multiple freeze-thaw cycles of frozen samples. Thaw completely and mix well prior to analysis.
7) When possible, avoid use of badly hemolyzed or lipemic serum. If large amounts of particulate matter are present, centrifuge or filter prior to analysis.
8) When pipetting reagents, maintain a consistent order of addition from well-to-well. This ensures equal incubation times for all wells.
9) Do not mix or interchange different reagent lots from various kit lots.
10) Do not use reagents after the kit expiration date.
11) Read absorbance immediately after adding the stop solution.

1. INTENDED USE

This TMAO ELISA kit is a 1.5 hour solid-phase ELISA designed for the quantitative determination of Human TMAO. This ELISA kit for research use only, not for therapeutic or diagnostic applications!

2. PRINCIPLE OF THE ASSAY

TMAO ELISA kit applies the competitive enzyme immunoassay technique utilizing a polyclonal anti-TMAO antibody and an TMAO-HRP conjugate. The assay sample and buffer are incubated together with TMAO-HRP conjugate in pre-coated plate for one hour. After the incubation period, the wells are decanted and washed five times. The wells are then incubated with a substrate for HRP enzyme. The product of the enzyme-substrate reaction forms a blue colored complex. Finally, a stop solution is added to stop the reaction, which will then turn the solution yellow. The intensity of color is measured spectrophotometrically at 450nm in a microplate reader. The intensity of the color is inversely proportional to the TMAO concentration since TMAO from samples and TMAO-HRP conjugate compete for the anti-TMAO antibody binding site. Since the number of sites is limited, as more sites are occupied by TMAO from the sample, fewer sites are left to bind TMAO-HRP conjugate. A standard curve is plotted relating the intensity of the color (O.D.) to the concentration of standards. The TMAO concentration in each sample is interpolated from this standard curve.

MyBioSource.com

* FOR RESEARCH PURPOSES ONLY. NOT FOR USE IN-DIAGNOSTIC PROCEDURES *

3. MATERIALS

All reagents provided are stored at 2-8° C. Refer to the expiration date on the label.

	MATERIALS	SPECIFICATION	QUANTITY
1	MICROTITER PLATE	96 wells	stripwell
2	ENZYME CONJUGATE	6.0 mL	1 vial
3	STANDARD A (0.5mL)	0 ng/mL	1 vial
4	STANDARD B (0.5mL)	5.0 ng/mL	1 vial
5	STANDARD C (0.5mL)	10 ng/mL	1 vial
6	STANDARD D (0.5mL)	25 ng/mL	1 vial
7	STANDARD E (0.5mL)	50 ng/mL	1 vial
8	STANDARD F (0.5mL)	100 ng/mL	1 vial
9	SUBSTRATE A	6 mL	1 vial
10	SUBSTRATE B	6 mL	1 vial
11	STOP SOLUTION	6 mL	1 vial
12	WASH SOLUTION (100 x)	10 mL	1 vial
13	BALANCE SOLUTION	3 mL	1 vial
14	INSTRUCTION	1	

NOTE: The BALANCE SOLUTION is used only when the sample is **cell culture supernatants, body fluid and tissue homogenate**; if the sample is serum or plasma, then the BALANCE SOLUTION is a superfluous reagent.

cross-reactivity detection between TMAO and all the analogues, therefore, cross reaction may still exist in some cases.

11. SAFETY NOTES

1) This kit contains small amount of 3, 3’, 5, 5’-Tetramethylbenzidine (TMB) in Substrate B. TMB is non-carcinogenic but it is hhazardous in case of skin contact, eye contact, ingestion and inhalation. In case of contact, rinse affected area with plenty of water.
2) The Stop Solution provided with this kit is an acid solution. Wear protective gloves, clothing, and face protection.
3) Care should be taken when handling the Standard because of the known and unknown effects of it.
4) Care should also be taken to avoid contact of skin or eyes with other kit reagents or specimens. In the case of contact, wash immediately with water.
5) Do not pipette by mouth.
6) Avoid generation of aerosols.
7) Waste must be disposed of in accordance with federal, state and local environmental control regulations.
8) All blood components and biological materials should be handled as potentially hazardous. Decontaminate and dispose specimens and all potentially contaminated materials as they could contain infectious agents. The preferred method of decontamination is autoclaving for a minimum of 1 hour a 121.5°C.

MyBioSource.com

* FOR RESEARCH PURPOSES ONLY. NOT FOR USE IN-DIAGNOSTIC PROCEDURES *

NOTE:

1) Any variation in operator, pipetting and washing technique, incubation time or temperature, and kit age can cause variation in result. Each user should obtain their own standard curve.
2) If samples have been diluted, the concentration read from the standard curve must be multiplied by the dilution factor.
3) If specimens generate values higher than the highest standard, dilute the specimens and repeat the assay.

10. CERTIFICATE OF ANALYSIS

1) In the same lot CV%:<10
2) Different lot CV%: <10
3) Spike Recovery: 92-101%
4) Linearity:

	Range %
1:1	94 – 102
1:2	92 - 104
1:4	98 - 105
1:8	94 - 109

5) Sensitivity: The sensitivity in this assay is 1.0 ng/mL.
6) Specificity: This assay has high sensitivity and excellent specificity for detection of TMAO. No significant cross-reactivity or interference between TMAO and analogues was observed. **NOTE**: Limited by current skills and knowledge, it is impossible for us to complete the

The types of sample:	
Sample I:	serum or plasma
Sample II:	cell culture supernatants, body fluid and tissue homogenate

4. SPECIMEN COLLECTION AND STORAGE

Serum - Use a serum separator tube and allow samples to clot for 2 hours at room temperature or overnight at 2-8°C. Centrifuge at approximately 1000 × g (or 3000 rpm) for 15 minutes. Collect serum and assay immediately or aliquot and store samples at -20°C or -80°C.

Plasma - Collect plasma using EDTA or heparin as an anticoagulant. Centrifuge samples for 15 minutes at 1000 × g (or 3000 rpm) at 2 - 8°C within 30 minutes of collection. Assay immediately or aliquot and store samples at -20°C or -80°C.

Tissue homogenates - The preparation of tissue homogenates will vary depending upon tissue type. For this assay, tissues were rinsed in ice-cold PBS (0.02mol/L, pH 7.0-7.2) to remove excess blood thoroughly and weighed 300-500mg before homogenization. Minced the tissues to small pieces and homogenized them in 500ul of PBS with a glass homogenizer on ice. The resulting suspension was subjected to ultrasonication or to two freeze-thaw cycles to further break the cell membranes. After that, the homogenates were centrifugated for 15 minutes at 1500×g (or 5000 rpm).

MyBioSource.com

* FOR RESEARCH PURPOSES ONLY. NOT FOR USE IN-DIAGNOSTIC PROCEDURES *

Collect the supernate and assay immediately or aliquot and store samples at -20°C or -80°C.

Cell lysates - Cells should be lysed according to the following directions.

1) Adherent cells should be detached with trypsin and then collected by centrifugation. Suspension cells can be collected by centrifugation directly.
2) Wash cells three times in PBS.
3) Cells were resuspended in PBS and subjected to ultrasonication for 3 times. Alternatively, freeze cells at -20 °C. Thaw cells with gentle mixing. Repeat the freeze/thaw cycle for 3 times.
4) Centrifuge at 1000×g (or 3000 rpm) for 15 minutes at 2-8 °C to remove cellular debris.
5) Assay immediately or store samples at -20°C or -80°C.

Cell culture supernatants and other body fluids - Centrifuge cell culture media at 1000 × g (or 3000 rpm) for 15 minutes to remove debris. Assay immediately or store samples at -20°C or -80°C.

NOTE:

1) Samples should be aliquoted and must be stored at -20°C (less than 3 months) or -80°C (less than 6 months) to avoid loss of bioactivity and contamination. If samples are to be run within 24 hours, they may be stored at 2- 8°C. Avoid repeated freeze-thaw cycles. Fresh samples without long time storage are recommended for the test. Otherwise, protein degradation and denaturalization may occur in those samples

1) The standard curve is used to determine the amount of samples.
2) First, average the duplicate readings for each standard and sample. All O.D. values are subtracted by the mean value of blank control before result interpretation. DO NOT subtract the O.D of standard zero.
3) Construct a standard curve by plotting the concentration on the horizontal (X) axis against the average O.D. for each standard on the vertical (Y) axis, and draw a best fit curve e using graph paper or statistical software to generate a four paramater logistic (4-PL) curve-fit or logit-log linear regression curve. An x-axis for the optical density and a y-axis for the concentration is also a choice. The data may be linearized by plotting the log of the concentrations versus the log of the O.D. and the best fit line can be determined by regression analysis.
4) Calculate the concentration of samples corresponding to the mean absorbance from the standard curve.
5) Standard curve for demonstration only.

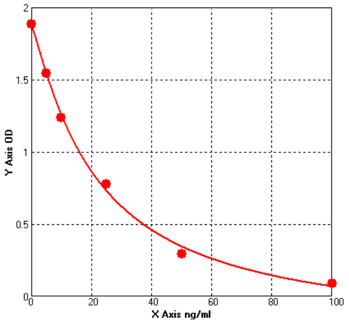

MyBioSource.com

* FOR RESEARCH PURPOSES ONLY. NOT FOR USE IN-DIAGNOSTIC PROCEDURES *

4) Wash the microtiter plate using one of the specified methods indicated below:

a) Manual Washing: Remove incubation mixture by aspirating contents of the plate into a sink or proper waste container. Fill in each well completely with 1× wash solution, and then aspirate contents of the plate into a sink or proper waste container. Repeat this procedure five times for a total of FIVE washes.

b) Automated Washing: Wash plate FIVE times with diluted wash solution (350-400 µL/well/wash) using an auto washer. It is recommended that the washer be set for a soaking time of 10 seconds and shaking time of 5 seconds between each wash.

5) After washing, invert plate, and blot dry by hitting the plate onto absorbent paper or paper towels until no moisture appears. Note: Hold the sides of the plate frame firmly when washing the plate to assure that all strips remain securely in frame. Complete removal of liquid at each step is essential to good performance.

6) Add 50 µL **Substrate A** and 50 µL **Substrate B** to each well including blank control well, subsequently. Cover and incubate for 15-20 minutes at 37°C. (Avoid sunlight If the color is not dark, please prolong the incubation time. But the longest time is 30min).

7) Add 50 µL of **Stop Solution** to each well including blank control well. Mix well.

8) Determine the Optical Density (O.D.) at 450 nm using a microplate reader immediately.

9. CALCULATION OF RESULTS

2) Tissue or cell extraction samples prepared by chemical lysis buffer may cause unexpected ELISA results due to the impacts of certain chemicals.

3) Samples containing a visible precipitate must be clarified prior to use in the assay. Care should be taken to minimize hemolysis. Do not use grossly hemolyzed or lipemic specimens.

4) Do not use heat-treated specimens.

5. MATERIALS AND EQUIPMENT REQUIRED BUT NOT SUPPLIED

1) Precision pipettors and disposable tips to deliver 10-1000 µl. A multi-channel pipette is desirable for large assays.
2) 100 mL and 1 liter graduated cylinders.
3) Distilled or deionized water.
4) Tubes to prepare sample dilutions.
5) Absorbent paper.
6) Microplate reader capable of measuring absorbance at 450 nm.
7) Centrifuge capable of 3000 × g.
8) Microplate washer or washing bottle.
9) Incubator (37°C).
10) Data analysis and graphing software.

6. SAMPLE PREPARATION

1) We are only responsible for the kit itself, but not for the samples consumed during the assay. The user should calculate the

MyBioSource.com

possible amount of the samples used in the whole test. Please reserve sufficient amount of samples in advance.

2) Please predict the concentration before assaying. If values for these are not within the range of the standard curve, **users must determine the optimal sample dilutions for their particular experiments**. We suggest pre-experimenting with neat (undiluted) samples, 1:2 or 1:4 dilutions. Please avoid diluting your samples more than 1:10 as it would exceed the dilution limit set for this kit. If the expected concentration of the target is beyond the detection range of the kit, please contact technical support.

3) If the samples are not indicated in the manual, a preliminary experiment to determine the validity of the kit is necessary.

4) Owing to the possibility of mismatching between antigen from other resource and antibody used in our kits (e.g., antibody targets conformational epitope rather than linear epitope), some native or recombinant proteins from other manufacturers may not be recognized by our products.

5) Influenced by the factors including cell viability, cell number and also sampling time, samples from cell culture supernatant may not be detected by the kit.

7. REAGENTS PREPARATION

1) Bring all kit components and samples to room temperature (20-25 °C) before use.

2) Samples - Please predict the concentration before assaying. If concentrations are unknown or not within the detection range, a preliminary experiment is recommended to determine the optimal

dilution. PBS (pH 7.0-7.2) or 0.9% physiological saline can be used as dilution buffer.

3) Wash Solution - Dilute 10 mL of Wash Solution concentrate (100×) with 990 mL of deionized or distilled water to prepare 1000 mL of Wash Solution (1×). If crystals have formed in the concentrate, warm to room temperature and mix gently until the crystals have completely dissolved. The 1× wash solution is stable for 2 weeks at 2-8°C.

4) Do not dilute the other components which are ready- to-use.

8. ASSAY PROCEDURE

Please read Reagents Preparation before starting assay procedure. It is recommended that all Standards and Samples be assayed in duplicate. It is strongly recommended to do a preliminary experiment before measuring all samples.

1) Secure the desired numbers of coated wells in the holder then add 100 µL of **Standards (**Shake the bottle of each standard gently by hand and Pipette up and down the solution of standard for 3 times before adding**)** or **Samples** to the appropriate well. Add 100 µL of PBS (pH 7.0-7.2) in the blank control well.

2) Dispense 10 µL of **Balance Solution** into 100 µL samples only, mix well. **(NOTE**: This step is required when the sample is cell culture supernatants, body fluid and tissue homogenate; if the sample is serum or plasma, then this step should be skipped.)

3) Add 50 µL of **Conjugate** to each well (NOT blank control well). Mix well. Mixing well in this step is important. Cover and incubate the plate for 1 hour at 37°C.

MyBioSource.com

* FOR RESEARCH PURPOSES ONLY. NOT FOR USE IN-DIAGNOSTIC PROCEDURES *

ELK Biotechnology

Human TMAO(Trimethylamine-N-oxide) ELISA Kit

Cat: ELK8356

96 Tests

For research use only. Not intended for diagnostic use.

Sensitivity: 0.065 μmol/L

Detection Range: 0.16-10 μmoL/L

Specificity: This assay has high sensitivity and excellent specificity for detection of Human TMAO. No significant cross-reactivity or interference between Human TMAO and analogues was observed.

Please refer to the outer packaging label of the kit for the specific shelf life.

KIT Components & Storage

Store kit at 4°C for 1 week. If the kit is not used up in 1 week, store the items separately according to the following conditions after the kit is received.

Reagents	Quantity		Storage Condition
	48T	96T	
Pre-Coated Microplate	6 strips x 8 wells	12 strips x 8 wells	-20°C (6 months)
Standard (Lyophilized)	1 vial	2 vials	-20°C (6 months)
Biotinylated Antibody (100×)	60 μL	120 μL	-20°C (6 months)
Streptavidin-HRP (100×)	60 μL	120 μL	-20°C (6 months)
Standard/Sample Diluent Buffer	10 mL	20 mL	4°C
Biotinylated Antibody Diluent	6 mL	12 mL	4°C
HRP Diluent	6 mL	12 mL	4°C
Wash Buffer (25×)	10 mL	20 mL	4°C
TMB Substrate Solution	6 mL	9 mL	4°C (store in dark)
Stop Reagent	3 mL	6 mL	4°C
Plate Covers	1 piece	2 pieces	4°C

Special Explanation

1. Please store kit at 4°C if used up in 1 week. If used for more than 1 week, store the Pre-Coated Microplate, Standard, Biotinylated Antibody and Streptavidin-HRP at -20°C and all other reagents at 4°C according to the temperature indicated on the label. Avoid repeated freeze-thaw cycles.
2. Do not use the kit beyond the expiration date.
3. After opening the package, please check that all components are complete.
4. The cap must be tightened to prevent evaporation and microbial contamination. The reagents volume is slightly more than the volume marked on labels, please use accurate measuring equipment and do not pour directly into the vial.

All kit components have been formulated and quality control tested to function successfully. Do not mix or substitute reagents or materials from other kits, detection effect of the kit will not be guaranteed if utilized separately or substituted.

Materials Required, Not Supplied

1. Microplate reader capable of measuring absorbance at 450 ± 10 nm.
2. High-speed centrifuge.
3. Electro-heating standing-temperature cultivator.
4. Absorbent paper.
5. Distilled or deionized water.
6. Single or multi-channel pipettes with high precision and disposable tips.
7. Precision pipettes to deliver 2 μL to 1 mL volumes.

Safety Notes

1. This kit is only used for lab research and development and should not be used for human or animals.
2. Reagents should be regarded as hazardous substances and should be handled carefully and correctly.
3. Gloves, lab coats, and goggles should always be worn to avoid skin and eyes coming into contact with Stop Solution and TMB. In case of contact, wash thoroughly with water.

Test Principle

The test principle applied in this kit is Sandwich enzyme immunoassay. The microtiter plate provided in this kit has been pre-coated with an antibody specific to Trimethylamine-N-oxide(TMAO). Standards or samples are added to the appropriate microtiter plate wells then with a biotin-conjugated antibody specific to Trimethylamine-N-oxide(TMAO). Next, Avidin conjugated to Horseradish Peroxidase (HRP) is added to each microplate well and incubated. After TMB substrate solution is added, only those wells that contain Trimethylamine-N-oxide(TMAO), biotin-conjugated antibody and enzyme-conjugated Avidin will exhibit a change in color. The enzyme-substrate reaction is terminated by the addition of sulphuric acid solution and the color change is measured spectrophotometrically at a wavelength of 450nm ± 10nm. The concentration of Trimethylamine-N-oxide(TMAO) in the samples is then determined by comparing the OD of the samples to the standard curve.

Sample Collection and Storage

Serum - Samples should be collected into a serum separator tube. After clotting for 2 hours at room temperature or overnight at 4°C, and then centrifuging at 1000 × g for 20 minutes. Assay freshly prepared serum immediately or store samples in aliquot at -20°C or -80°C for later use. Avoid repeated freeze-thaw cycles.

Plasma - Collect plasma using EDTA or heparin as an anticoagulant. Centrifuge samples at 1000 × g and 2-8°C for 15 minutes within 30 minutes of collection. Remove plasma and assay immediately or store samples in aliquot at -20°C or -80°C for later use. Avoid repeated freeze-thaw cycles.

Tissue homogenates - The preparation of tissue homogenates will vary depending upon tissue type.

1. Tissues were rinsed in pre-cooled PBS to remove excess blood thoroughly and weighed before homogenization.
2. Minced the tissues to small pieces and homogenized them in fresh lysis buffer (different lysis buffer needs to be chosen based on subcellular location of the target protein) (PBS can be used as the lysis buffer of most tissues) (w:v = 1:9, e.g. 900 μL lysis buffer is added in 100 mg tissue sample) with a glass homogenizer on ice (micro tissue grinders, too).
3. The resulting suspension was sonicated with an ultrasonic cell disrupter till the solution was clarified.
4. Then, the homogenates were centrifuged for 5 minutes at 10000 × g and collect the supernatant and assay immediately or aliquot and store at ≤ -20°C.

*Note: Tissue homogenate samples are recommended to be tested for protein concentration at the same time to obtain a more accurate concentration of the test substance per mg of protein. For protein detection, you can purchase our product: **BC016, BCA Protein concentration determination kit**.

Cell lysates - Cells need to be lysed before assaying according to the following directions.

1. Adherent cells should be washed by pre-cooled PBS gently, and then detached with trypsin, and collected by centrifugation at 1000 × g for 5 minutes (suspension cells can be collected by centrifugation directly).
2. Wash cells three times in pre-cooled PBS.
3. Cells were then resuspended in fresh lysis buffer with concentration of 10^7 cells/mL. If it was necessary, the cells could be subjected to ultrasonication till the solution is clarified.

4. Centrifuge at 1500 × g for 10 minutes at 2-8°C to remove cellular debris. Assay immediately or aliquot and store at ≤ -20°C.

Urine - Aseptically collect the first urine of the day (mid-stream), voided directly into a sterile container. Centrifuge to remove particulate matter, assay immediately or aliquot and store at ≤-20°C.Avoid repeated freeze-thaw cycles.

Saliva - Collect saliva using a collection device or equivalent. Centrifuge samples for 15 minutes at 1000 × g at 2-8°C. Remove particulates and assay immediately or store samples in aliquot at ≤ -20°C. Avoid repeated freeze-thaw cycles.

Cell culture supernatants and other biological fluids - Centrifuge samples for 20 minutes at 1000 × g. Collect the supernatant and assay immediately or store samples in aliquot at -20°C or -80°C for later use. Avoid repeated freeze-thaw cycles.

Notes

1. Samples to be used within 5 days may be stored at 4°C, otherwise samples must be stored at -20°C (≤ 1 month) or -80°C (≤ 2 months) to avoid loss of bioactivity and contamination. Avoid repeated freeze-thaw cycles.
2. Sample hemolysis will influence the result, so hemolytic specimen should not be used.
3. When performing the assay, bring samples to room temperature.
4. If the concentration of the test material in your sample is higher than that of the Standard product, please make the appropriate multiple dilutions according to the actual situation (it is recommended to do preliminary experiment to determine the dilution ratio.

Summary

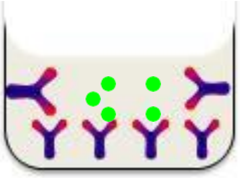

1. After the kit is equilibrated at room temperature, add 100 μL of Standard Working Buffer (gradually diluted according to the instructions) or 100 μL of sample to each well, and incubate at 37°C for 80 minutes.

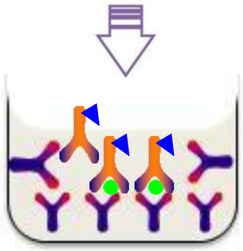

2. Discard the liquid in the plate, add 200 μL of 1×Wash Buffer to each well, and wash the plate 3 times. After spin-drying, add 100 μL Biotinylated Antibody Working Solution (1×) to each well, incubate at 37°C for 50 minutes.

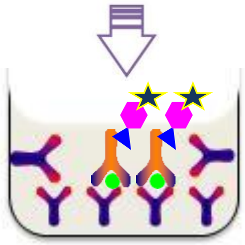

3. Discard the liquid in the plate, add 200 μL 1 ×Wash Buffer to each well, and wash the plate 3 times. After drying, add 100 μL 1×Streptavdin-HRP Working Solution to each well, incubate at 37°C for 50 minutes.

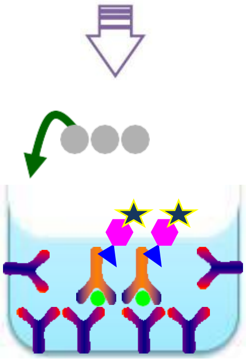

4. Discard the liquid in the plate, add 200 μL 1×Wash Buffer to each well, and wash the plate 5 times. After spin-drying, add 90 μL TMB Substrate Solution to each well, incubate at 37°C for 20 minutes in the dark.

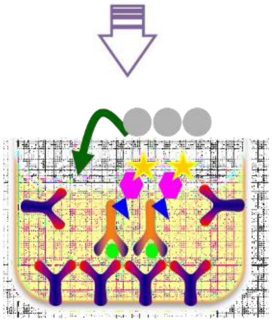

5. Add 50 μL Stop Solution to each well, shake plate on a plate shaker for 1 minute to mix. Record the OD at 450 nm immediately, calculation of the results.

Reagent Preparation

1. Bring all kit components and samples to room temperature (18-25°C) before use.
2. If the kit will not be used up in one time, please only take out strips and reagents for present experiment, and save the remaining strips and reagents as specified.
3. Dilute the 25× Wash Buffer into 1× Working Concentration with Double-distilled Water.
4. **Standard Working Solution** - Centrifuge the Standard at 1000 × g for 1 minute. Reconstitute the Standard with 1.0 mL of Standard Diluent Buffer, kept for 10 minutes at room temperature, shake gently (not to foam). The concentration of the Standard in the stock solution is 10 μmoL/L. Please prepare 7 tubes containing 0.5 mL Standard Diluent Buffer and use the Diluted Standard to produce a double dilution series according to the picture shown below. To mix each tube thoroughly before the next transfer, pipette the solution up and down several times. Set up 7 points of Diluted Standard such as 10 μmoL/L, 5 μmoL/L, 2.5 μmoL/L, 1.25 μmoL/L, 0.63 μmoL/L, 0.32 μmoL/L, 0.16 μmoL/L, and the last EP tubes with Standard Diluent is the Blank as 0 μmoL/L. In order to guarantee the experimental results validity, please use the new Standard Solution for each experiment. When diluting the Standard from high concentration to low concentration, replace the pipette tip for each dilution. Note: the last tube is regarded as a Blank and do not pipette solution into it from the former tube.

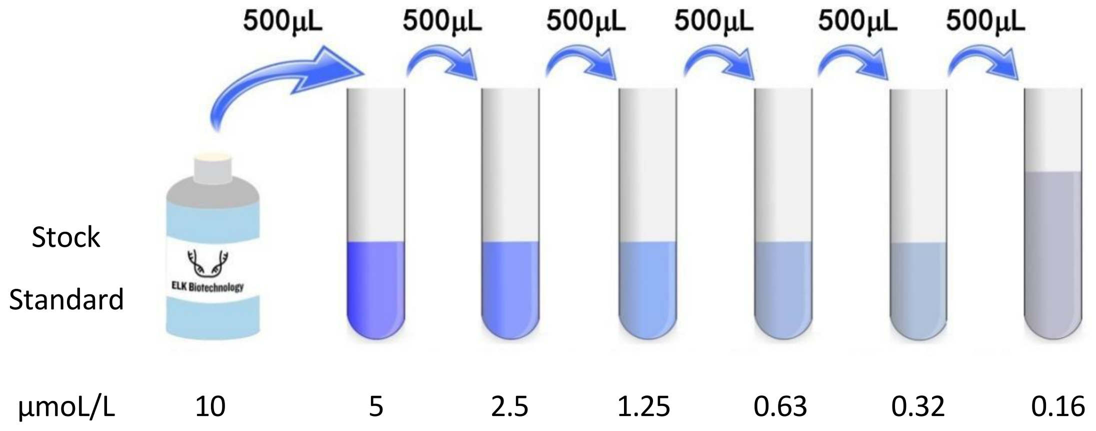

5. **Biotinylated Antibody and Streptavidin-HRP:** Briefly spin or centrifuge the stock Biotinylated Antibody and Streptavidin-HRP before use. Dilute them to the working concentration 100-fold with Biotinylated Antibody Diluent and HRP Diluent, respectively.
6. **TMB Substrate** - Aspirate the needed dosage of the solution with sterilized tips and do not dump the

residual solution into the vial again.

Notes

1. After receive the kit, please store the reagents according to the instructions. The plates can be disassembled to single strips. Please use it in batches on demands. It is recommended that the remaining reagents are used within 1 month after the first test.
2. The test tubes, pipette tips and reagents used in the experiment are all disposable and are strictly prohibited from being reused; otherwise the experiment results will be affected. Kit reagents of different batches cannot be mixed (except TMB, Washing Buffer and Stop Reagent).
3. Lyophilized Standards, Biotinylated Antibody, and Streptavidin-HRP are small in volume and may be scattered in various parts of the tube during transportation. Please centrifuge at 1000 × g for 1 minute before use to allow the liquid on the tube wall or bottle cap to settle to the bottom of the tube. Before use, carefully pipette 4-5 times to mix the Solution. Please configure the Standard, Biotinylated Antibody and Streptavidin-HRP Working Solution according to the required amount, and use the corresponding Dilution Solution, cannot be mixed used.
4. Bring all reagents to room temperature (18-25°C) before use. If crystals form in the concentrate (25 ×), it is a normal phenomenon. Heat it to room temperature (the heating temperature should not exceed 40°C), gently Mix until crystals are completely dissolved.
5. Prepare to dissolve Standard within 15 minutes before the test. This Standard Working Solution can only be used once. If the dissolved Standard is not used up, please discard it. The sample addition needs to be rapid. Each sample addition should preferably be controlled within 10 minutes. To ensure experimental accuracy, it is recommended to test duplicate wells, and when pipetting reagents, keep a consistent order of additions from 1 well to another, this will ensure the same incubation time for all wells.
6. During the washing process, the residual washing liquid in the reaction well should be patted dry on absorbent paper. Do not put the paper directly into the reaction well to absorb water. Before reading, pay attention to remove the residual liquid and fingerprints at the bottom, so as not to affect the microplate reader reading.
7. TMB is light-sensitive, avoid prolonged exposure to light. Dispense the TMB Solution within 15 minutes following the washing of the microtiter plate. In addition, avoid contact between TMB

Solution and metal to prevent color development. TMB is contaminated if it turns blue color before use and should be discarded. TMB is toxic, avoid direct contact with hands.

8. Bacterial or fungal contamination of either samples or reagents or cross-contamination, between reagents may cause erroneous results.

Samples Preparation

1. Equilibrate all materials and prepared reagents to room temperature prior to use. Prior to use, mix all reagents thoroughly taking care not to create any foam within the vials.
2. The user should calculate the possible amount of the samples used in the whole test. Please reserve sufficient samples in advance.
3. Please predict the concentration before assaying. If values for these are not within the range of the Standard curve, users must determine the optimal sample dilutions for their particular experiments.

Assay Procedure

1. Determine wells for Diluted Standard, Blank and Sample. Prepare 7 wells for Standard, 1 well for Blank. Add 100 μL each of Standard Working Solution (please refer to **Reagent Preparation**), or 100 μL of samples into the appropriate wells. Cover with the Plate sealer. Incubate for 80 minutes at 37°C. Note: solutions should be added to the bottom of the micro ELISA plate well, avoid touching the inside wall and causing foaming as much as possible.
2. Remove the liquid of each well. Aspirate the solution and wash with 200 μL of 1 × Wash Solution to each well and let it sit for 1-2 minutes. Remove the remaining liquid from all wells completely by snapping the plate onto absorbent paper. Totally wash 3 times. After the last wash, remove any remaining Wash Buffer by aspirating or decanting. Invert the plate and blot it against absorbent paper.

   Notes: (a) When adding Washing Solution, the pipette tip should not touch the wall of the wells to a void contamination.

   (b) Pay attention to pouring the washing liquid directly to ensure that the washing liquid does not contaminate other wells.
3. Add 100 μL of Biotinylated Antibody Working Solution to each well, cover the wells with the plate sealer and incubate for 50 minutes at 37°C.

4. Repeat the aspiration,wash process for total 3 times as conducted in step 2.
5. Add 100 μL of Streptavidin-HRP Working Solution to each well, cover the wells with the plate sealer and incubate for 50 minutes at 37°C.
6. Repeat the aspiration, wash process for total 5 times as conducted in step 2.
7. Add 90 μL of TMB Substrate Solution to each well. Cover with a new Plate sealer. Incubate for 20 minutes at 37°C (Don't exceed 30 minutes). Protect from light. The liquid will turn blue by the addition of TMB Substrate Solution. Preheat the Microplate Reader for about 15 minutes before OD measurement.
8. Add 50 μL of Stop Reagent to each well. The liquid will turn yellow by the addition of Stop Reagent. Mix the liquid by tapping the side of the plate. If color change does not appear uniform, gently tap the plate to ensure thorough mixing. The insertion order of the Stop Reagent should be the same as that of the TMB Substrate Solution.
9. Remove any drop of water and fingerprint on the bottom of the plate and confirm there is no bubble on the surface of the liquid. Then, run the microplate reader and conduct measurement at 450 nm immediately.

Calculation of Results

Average the duplicate readings for each Standard, Control, and Samples and subtract the average zero Standard optical density. Construct a Standard curve with the Human TMAO concentration on the y-axis and absorbance on the x-axis, and draw a best fit curve through the points on the graph. If samples have been diluted, the concentration read from the Standard curve must be multiplied by the dilution factor. Using some plot software, for instance, curve expert.

Concentration (μmoL/L)	OD	Corrected OD
10	2.256	2.168
5	1.628	1.54
2.5	1.216	1.128
1.25	0.752	0.664
0.63	0.509	0.421
0.32	0.265	0.177
0.16	0.196	0.108
0	0.088	0.000

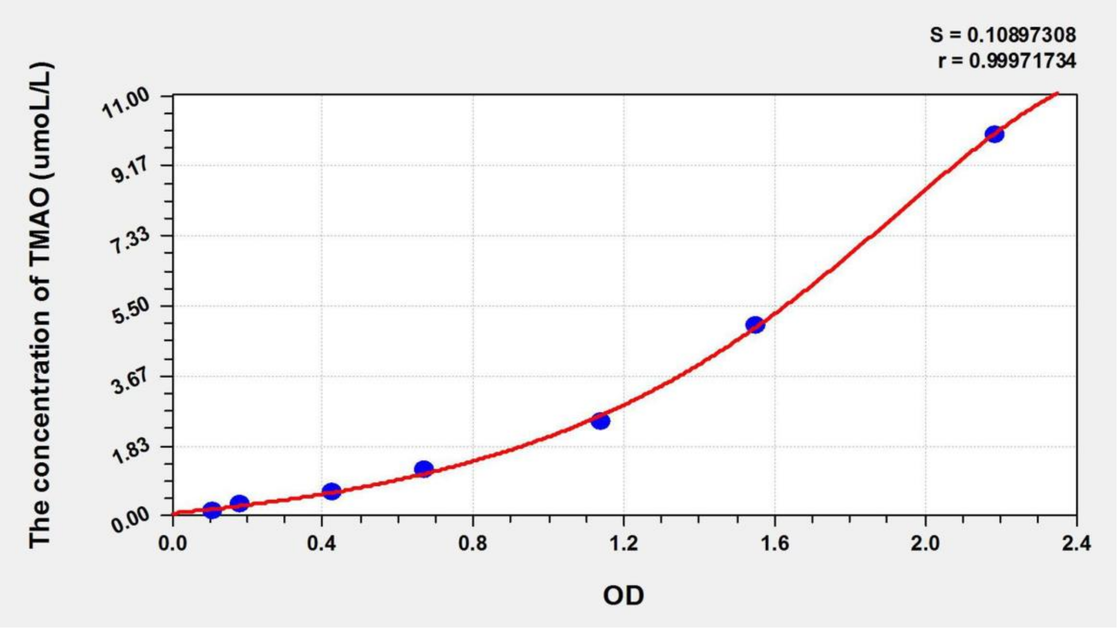

Note: this graph is for reference only

Precision

Intra-assay Precision (Precision within an assay)： **CV% < 8%**

Three samples of known concentration were tested twenty times on 1 plate to assess intra-assay precision.

Inter-assay Precision (precision between assays)： **CV% < 10%**

Three samples of known concentration were tested in forty separate assays to assess inter-assay precision.

Recovery

Matrices listed below were spiked with certain level of recombinant TMAO and the recovery rates were calculated by comparing the measured value to the expected amount of TMAO in samples.

Matrix	Recovery range	Average
Serum (*n* = 5)	83-97%	90%
EDTA plasma (*n* = 5)	87-95%	91%
Heparin plasma (*n* = 5)	81-95%	88%

Linearity

The linearity of the kit was assayed by testing samples spiked with appropriate concentration of TMAO and their serial dilutions. The results were demonstrated by the percentage of calculated concentration to the expected.

Sample	1:2	1:4	1:8	1:16
Serum (*n* = 5)	88-97%	86-92%	81-93%	93-104%
EDTA plasma (*n* = 5)	87-94%	91-104%	95-107%	82-96%
Heparin plasma (*n* = 5)	81-94%	93-107%	93-102%	91-105%

Declaration

1. The kit may not be suitable for special experimental samples where the validity of the experiment itself is uncertain, such as gene knockout experiments.
2. Certain natural or recombinant proteins, including prokaryotic and eukaryotic recombinant proteins, may not be detected because they do not match the detection antibody and capture antibody used in this product.
3. This kit is not compared with similar kits from other manufacturers or products with different methods to detect the same object, so inconsistent test results cannot be ruled out.

Analysis of Common Problems and Causes of ELISA Experiment

High background/non-specific staining

Description of results	Possible reason	Recommendations and precautions
After termination, the whole plate results show a uniform yellow or light color; or the Standard curve is linear but the background is too high	The yellowing of the whole plate may be caused by wrong addition of other reagents	Check the components and lot numbers of the reagents before the experiment, and confirm that all components belong to the corresponding kit. Reagents from different kits or different lot numbers cannot be mixed.
	ELISA plate was not washed sufficiently	Make sure that the same amount of Washing Solution is added to each microwell during the washing process. After washing, press the ELISA plate firmly on the absorbent paper to remove the residual buffer.
	Incubation time too long	Please strictly follow the steps of the manual
	Streptavidin-HRP contaminates the tip and TMB container or positive control contaminates the Pre-coated Microplate	When absorbing different reagents, the tips should be replaced. When configuring different reagent components, different storage vessels should be used. Please use a pipette during operation.
	Biotinylated Antibody or Streptavidin-HRP concentration too high	Check whether the concentration calculation is correct or use after further dilution.
	Substrate exposure or contamination prior to use	Store in the dark at all times before adding substrate.
	Color development time is too long	Please strictly follow the steps of the manual.
	The wrong filter was used	When TMB is used as the substrate, the

	when the absorbance value was read	absorbance should be read at 450 nm.

NO color plate

Description of results	Possible reason	Recommendations and precautions
After the color development step, all wells of the ELISA plate are colorless; the positive control is not obvious	Mixed use of component reagents	Please read labels clearly when preparing or using
	In the process of plate washing and sample addition, the enzyme marker is contaminated and inactivated, and loses its ability to catalyze the color developing agent	Confirm that the container holding the ELISA plate does not contain enzyme inhibitors (such as NaN_3, etc.), and confirm that the container for preparing the Wash Solution has been washed.
	Missing a reagent or a step	Review the manual in detail and strictly follow the operating steps

Light color

Description of results	Possible reason	Recommendations and precautions
The Standard is normal, the color of the sample is light	The sample uses NaN_3 preservative, which inhibits the reaction of the enzyme	Samples cannot use NaN_3
	The sample to be tested may not contain strong positive samples, so the result may be normal	In case of doubt, please test again.
The visual result is normal, but the reading value of the microplate reader is low	Wrong filter used for absorbance reading	When TMB is used as the substrate, the absorbance should be read at 450 nm.

Description of results	Possible reason	Recommendations and precautions
All wells, including Standard and Samples, are lighter in color	Insufficient incubation time	Timer accurate timing
	Insufficient color reaction	Usually 15 - 30 minutes
	The number of washings increases, and the dilution ratio of the concentrated lotion does not meet the requirements	Reduce the impact of washing, dilute the concentrated lotion and washing time according to the manual, and accurately record the washing times and dosage.
	Distilled water quality problem	The prepared lotion must be tested to see if the pH value is neutral.
	In the process of plate washing and sample addition, the enzyme marker is contaminated and inactivated, and loses its ability to catalyze the color developing agent.	Confirm that the container holding the ELISA plate does not contain enzyme inhibitors (such as NaN_3, etc.), confirm that the container for preparing the Washing Solution has been washed, and confirm that the purified water for preparing the Washing Solution meets the requirements and is not contaminated.
	The kit has expired or been improperly stored	Please use it within the expiration and store it in accordance with the storage conditions recommended in the manual to avoid contamination.
	Reagents and samples are not equilibrated before use	All reagents and samples should be equilibrated at room temperature for about 30 minutes.
	Insufficient suction volume of the pipette, too fast discharge of pipetting suction, too much liquid hanging on the inner wall of the tip or the inner wall is not clean.	To calibrate the pipette, the tips should be matched, each time the tips should fit tightly, the pipetting should not be too fast, and the discharge should be complete. The inner wall of the tips should be clean, and it is best to use it once.

Description of results	Possible reason	Recommendations and precautions
Poor repeatability	Incubation temperature constant temperature effect is not good	Keep the temperature constant to avoid the local temperature being too high or too low
	When adding liquid, too much remains on the medial wall of wells	When adding liquid, the tip should try to add liquid along the bottom of the medial wall of wells without touching the bottom of the hole.
	Reuse of consumables	The tips should be replaced when different reagents are drawn, and different storage vessels should be used when configuring different reagent components.
	The bottom of the microwell is scratched or there is dirt	Be careful when operating, be careful not to touch the bottom and wipe the bottom of the microplate to remove dirt or fingerprints.
		Technical repetition of the same sample for 3 times, including more than 2 approximate values.
	Cross-contamination during sample addition	Try to avoid cross-contamination when adding samples
The color of plate is chaotic and irregular	Cross-contamination from manual plate washing	When washing the plates by hand, the first 3 injections of the lotion should be discarded immediately, and the soaking time should be set for the next few times to reduce cross-contamination.
	Cross-contamination when clapping	Use a suitable absorbent paper towel when clapping the plate, do not pat irrelevant substances into the well of the plate, and try not to pat in the same position to avoid cross-contamination.

Description of results	Possible reason	Recommendations and precautions
The color of plate is chaotic and irregular	The liquid filling head of the plate washer is blocked, resulting in unsatisfactory liquid addition or large residual amount of liquid suction, resulting in the color of plate is chaotic and irregular	Unblock the liquid addition head, so that each well is filled with washing liquid when washing the plate and the residual amount should be small when aspirating liquid.
	Incomplete centrifugation of the sample, resulting in coagulation in the reaction well or interference of sediment or residual cellular components	Serum plasma should be fully centrifuged at 3000 rpm for more than 6 minutes
	The sample is stored for too long time, resulting in contamination.	Samples should be kept fresh or stored at low temperature to prevent contamination
	Incorrect preparation of Washing Solution or direct misuse of concentrated Washing Solution	Please configure according to the manual
